# Antibody Drug Conjugate Bioinformatics: Drug Delivery through the Letterbox

**DOI:** 10.1155/2013/282398

**Published:** 2013-06-19

**Authors:** Dimitrios Vlachakis, Sophia Kossida

**Affiliations:** Bioinformatics & Medical Informatics Team, Biomedical Research Foundation, Academy of Athens, Soranou Efessiou 4, 11527 Athens, Greece

## Abstract

Antibodies appear to be the first line of defence in the adaptive immune response of vertebrates and thereby are involved in a multitude of biochemical mechanisms, such as regulation of infection, autoimmunity, and cancer. It goes without saying that a full understanding of antibody function is required for the development of novel antibody-interacting drugs. These drugs are the Antibody Drug Conjugates (ADCs), which are a new type of targeted therapy, used for example for cancer. They consist of an antibody (or antibody fragment such as a single-chain variable fragment [scFv]) linked to a payload drug (often cytotoxic). Because of the targeting, the side effects should be lower and give a wider therapeutic window. Overall, the underlying principle of ADCs is to discern the delivery of a drug that is cytotoxic to a target that is cancerous, hoping to increase the antitumoural potency of the original drug by reducing adverse effects and side effects, such as toxicity of the cancer target. This is a pioneering field that employs state-of-the-art computational and molecular biology methods in the fight against cancer using ADCs.

## 1. Introduction

Antibodies, or immunoglobulins, belong to the “gamma globulin” protein group and can be found mainly in the blood of vertebrates [1]. Antibodies constitute the major serological line of defense of the vertebrates with jaws (gnathostomata) by which the immune system identifies and neutralizes threatening invaders, such as viruses, fungi, parasites, and bacteria. The contrivance underlying the reaction efficiency of our immune system to specifically recognize and fight invading organisms or to trigger an autoimmune response and disease still remains to be elucidated.

The efficient reaction of our immune system against all kinds of intruders is highly dependent on the number, condition, and availability of antibodies, as reaction times are “key” to the successful elimination of the foreign pathogen. On the other hand, antibodies can be described as an inappropriate and offensive response of the immune system against normal tissues of the body. In essence the immune system mistakenly recognizes its own cells as potential pathogens and attacks them. In most cases this reaction may be localized on just parts of certain organs or include a specific type of tissue that can be found in more than one organ in the human body. Up until now, the most commonly practiced clinical treatments for diseases of the immune system involve immunosuppression, which aims to lessen the reactive immune response.

An antibody is made up of two identical heavy chains (H) and two identical light chains (L) with for each one, variable (V) domain at the N-terminal end [1]. Antibodies contain variable domains characterized by structurally hypervariable regions, also known as complementarity determining regions (CDRs), which allow them to recognize an equally diverse number of antigens [2]. The recognition site is made up by the CDRs, three per domain (CDR1, CDR2, and CDR3). As the CDR3 results from the rearrangement of three genes (variable (V), diversity (D), and joining (J)) for the heavy chain, of two genes (V, J) for the light chain kappa or lambda, this creates a huge diversity of antibodies (10^12^ per individual, the limiting factor being only the number of B cells that an organism can genetically produce). These antibodies have the capability of recognizing a similarly huge number of antigens. The three CDRs are responsible for the structural interaction between the antibody variable domains and the antigen shape and size. In essence the CDRs dictate the antibody specificity and affinity for a specific antigen. 

A paratope is the antibody region responsible for interacting with the corresponding epitope region of an antigen. The recognition sites of the antibody and the antigen allow the two molecules to structurally form a complex conformation. It is through this binding interaction that antibodies tag invaders that must be either neutralized or eliminated. Specificity is an important property of antibody as it refers to the ability of an individual antibody (or of its clonal population) to specifically recognize and bind to a specific antigenic determinant. 

## 2. Importance of Antibody Drug Conjugate (ADC) Technology

The importance of antibodies in health care and the biotechnology industry demands knowledge of their structures at high resolution. This information can be used for antibody engineering, modification of the antigens binding affinity, and epitope identification of a given antibody. Computational approaches provide a cheaper and faster alternative to the commonly used, albeit laborious and time consuming, X-ray crystallography. Available immunogenetics data can be used for computational modelling of antibody variable domains. Standardized amino acid positions and properties can assist in optimizing the relative orientation of light and heavy chains as well as in designing homology models that predict successful docking of antibodies with their unique antigen.

Towards this direction, the international ImMunoGeneTics information system (IMGT, http://www.imgt.org) in Montpellier, France, has built an ontology (IMGT-ONTOLOGY) from which novel concepts and standards for immunogenetics and immunoinformatics are generated, making IMGT the global reference in the domain. Using starting material from the IMGT antibody database (i.e., human antibodies), novel *de novo* structure based drug design techniques are being applied in order to develop Antibody Drug Conjugates (ADCs) as potent anticancer agents. The ADC technology basically involves three parts: the antibody-carrier, the linker, and the drug. The main focus of this project is the development of versatile, highly efficient linker molecules that will be used to fuse the chemotherapeutic agent onto the carrier antibody. Linkers should be able to be tolerant and robust during preparation, administration, and circulation of the ADC and be highly efficient in getting cleaved by host enzymes only in the host-target cell's cytoplasm. This way, a series of chemotherapeutic agents is tested for more targeted anticancer therapies.

## 3. Why Study and Model Antibodies for ADCs?

The need for new therapeutic targets and for the development of more potent anticancer drugs is enormous, despite the efforts and investments for the past few years for the development of novel anticancer drugs and the discovery and characterization of new therapeutic targets using antibodies [3]. It is well established that the diverse biological functions of proteins are dependent upon their three-dimensional structure, which in turn is determined by their primary amino acid sequence. 

There is significant biotechnological and medical interest in developing novel drugs acting on new target molecules. However, even though a series of quite potent chemotherapeutic agents has already been developed, tackling cancer is still let down by their side effects. All drugs are eventually failing due to their inability to recognize only the cancer cell and not harm normally proliferating host cells. Therefore, antibodies can be used as drug carriers that will deliver the chemotherapeutic agent with great accuracy to the cancerous cell only. A rational approach towards this goal is the design of linker molecules and anticancer agents based on the detailed 3D structure of their potential protein target, an approach becoming common among pharmaceutical companies [4]. Therefore, knowledge of the detailed dynamic 3D structure of antibodies is fundamental for fully understanding its role in defence and autoimmunity, which is a necessary prerequisite for the rational development of novel antibody-based drugs. 

Until today, a multitude of three-dimensional structures of antibodies have been determined. However, the structural basis of their interactions remains to be elucidated [5]. 3D modelling of antibodies requires prior knowledge of the 3D structure of similar protein(s) and is based on the observation that proteins sharing sequence similarities also follow similar folds. This approach is known as comparative modelling [6]. On the other hand, even unrelated sequences have been found to follow similar folds. The methodology used to predict the three-dimensional structure in this case is called threading because it involves threading a specific sequence through all known folds and estimating of the energetics of the resulting structures. 

## 4. Development of Antibody Drug Conjugates

As described previously, antibodies will bind to a huge number of targets with high affinity and specificity. Five distinct classes of antibodies have been identified in humans (IgA, IgM, IgD, IgE, and IgG). The trait is that antibodies in each group have specific physicochemical properties and share similar roles in immune response [7]. However, structure is far more conserved than sequence in nature. Thereof, structural analysis and comparison amongst proteins in the antibody realm are bound to yield much more reliable data and information.

To date, there have been major applications of antibodies, against diseases such as rheumatoid arthritis, leukemia, multiple sclerosis, and cancer with very promising outcomes [8, 9]. Moreover, using xenogeneic antibodies has also been found to reduce drug effectiveness due to their immunogenicity. As a result, 3D molecular modelling, protein engineering, and structural bioinformatics have been employed in an effort to modify and to manipulate the spatial dynamics and arrangement of antibody molecules. This way, the molecular interactions towards the partner, interacting molecule may be altered alongside the antibodies physicochemical composition, hydropathy, half-life, effector function, its immunogenicity, and binding affinity. State-of-the-art antibody protein engineering has been used to exchange/swap parts of interest among different antibodies or even to attach other molecular structures on antibodies. The latter gave rise to a new clan of pioneering research methods that work on the futuristic field of Antibody Drug Conjugates (ADCs). Antibody Drug Conjugates are a new type of targeted therapy that has already been successfully used for cancer [10–13]. They consist of an antibody (or antibody fragment such as a single-chain variable fragment [scFv]) linked to a payload drug (often cytotoxic) [14]. Henceforth, there are examples of bioconjugates and immune-conjugates that often bear a toxin. The mechanism behind this is that the antibody itself drives the ADC complex to bind to the target cancer cell, where the latter is then internalized, into the cell. Inside the cancer cell, the drug is detached and is released to do its function. The role of such drugs is to inflict damage to the cell. Due to the elevated detecting abilities of the cell targeting, using the antibody Trojan horse, the toxin side effects are lowered and provide a wider, more diverse, therapeutic window [15]. Homology and comparative modelling have been widely used to determine the unknown 3D structures of antibodies, based on their primary amino acid composition, so that they may be used as the scaffold on which ADC technology will be applied towards novel, humanized, and drug-like molecular complexes, based on antibodies [16]. The current chemotherapeutic mechanism of conventional drug action is linked to the increased proliferating rates of cancer cells when compared to normal ones. Nevertheless, this conventional approach is far from being specific, as it is very unselective to the cell types it attacks and may prove to be highly toxic to healthy cells that normally have high metabolic rates, for instance, cells of the bone marrow, the gastrointestinal tract, and the most prominent hair follicles, which explains why patients during chemotherapy lose their hair. Therefore each chemotherapeutic is only limited in dose by the severity of the side effects it induces. In most cases suboptimal doses are only allowed and the success of the chemotherapy session is compromised. Linking a tumour-specific antibody to a potent chemotherapeutic agent is a novel approach that bears great promises in more accurate cancer targeting. Chemically, Antibody Drug Conjugates are made up of an internalizing tumour-targeting mAb that is covalently linked with a chemotherapeutic agent using an organic biochemical linker or aliphatic chain. The linking moiety must be stable enough to withstand normal blood circulation and to be precisely cleaved upon specific cell-uptake. This way it will release the drug into the cancer cell straight from the antibody. The ratio of the antibody to drug copies is very important. If many copies of a drug can be attached onto a single antibody molecule that is still functional, then the drug does not have to be very potent. This is quite rare though as to date there are no more than four copies of a drug attached to a carrier antibody, and that requires that the drug must be highly potent or highly toxic to the cancer cell. A major bottleneck for the ADC technique is the reduced tumour uptake combined with a relative antigenic heterogeneity and normal tissue toxicity. Specifically liver tissue seems to suffer greatly from current ADC complexes in preclinical studies, since it is responsible for processing large macromolecules from the blood stream and that includes antibodies too. However, very little is known in this direction as to date there is only a single ADC on the pharmaceutical market. It is called Mylotarg against acute myeloid leukaemia [17]. A humanized anti-CD33 antibody is linked to the calicheamicin molecule, which is a low molecular weight, highly potent drug toxin. Rational drug design, commonly employed by many pharmaceutical companies, involves first identification of new drug targets followed by determination of their 3D structure. Knowledge of the detailed 3D structure of protein targets can be used for the *in silico* design of novel compounds that can alter the targets function as desired [18]. Such new compounds can thus be the basis for the development of new drugs against pathologies related to the function of the protein targets. Although our knowledge of antibody function is at present well established, its role in evader recognition and biochemical or structural modifications they undergo makes them a strong candidate for future rational drug design approaches. Information obtained by the proposed combined functional and structural studies will be invaluable to determine whether antibodies could indeed be a pharmaceutical target for “structure-based drug design” approaches [19]. 

There is a small variety of different conjugation methods that are employed for the production of computer-engineered ADC agents. These specific methodologies include enzymatic conjugation, targeting of lysine and cysteine residues, and glycoengineering. Enzymatic conjugation is mainly focused on the exploitation of the enzymatic activity of neuraminidase, which is used for the coupling of the doxorubicin antineoplastic agent [20]. Four copies of the drug were loaded onto the antibody. *In silico *study could be very useful here in an effort to optimize the structural releasing features of the linker molecules. Then there is the targeting of the lysine and the cysteine residues. A set of highly optimized computer algorithms have already been developed for the identification of such key-target residues, which are suitable as anchoring moieties on the antibody. The criteria generally are that either the lysine or the cysteine residue has to be exposed to the solvent and thereof reactive and far away from the complementarity determining regions (CDRs) of the antibody. This is very important as targeting residues in the proximity of the CDRs could have a negative effect, inhibiting the interaction of the antibody with its target host cell epitope. An average antibody has a total of approximately ninety lysine residues, while at the same time it has less than ten cysteine residues. Our *in silico *tool will recognize each one of those potential target residues, will isolate the exposed ones that are not already in some form of intramolecule interaction (such as disulphide bridges), and will return them to the user as potential anchoring points for the drug design experiment. Finally glycoengineering involves the biotinylation of mAbs with the biotinyl-N-hydroxysuccinimide ester. O'Shannessy et al. managed to develop a new approach that leads to more specific biotinylation [21]. Bioinformatics software can be used to predict biotinylation sites on antibodies towards the development of ADCs.

## 5. Conclusion

The ultimate goal of today's antibody research is to provide insights into antibody/antigen specific interaction properties and the *de novo* development of Antibody Drug Conjugates (ADCs) by carefully investigating a series of CDR sequences using multidisciplinary approaches. Vast knowledge on antibody engineering and chemotherapy is used in an effort to develop optimized, highly efficient linker molecules that will release the drug only in the specific (cancerous) cell, thus reducing cytotoxicity and adverse effect induced by the anticancer agent. 

ADCs are made up by the cytotoxic drug payload, the linker molecule, and the targeting carrier antibody. 

Extra effort is invested in simulating antibody overloading techniques with drug molecules in an effort to achieve higher efficacy. The payload drugs include several toxins with different mechanism of action, intercalating agents, microtubule binders, topoisomerase I inhibitors, and DNA binders to minor grooves. The linker molecule will either be uncleavable or cleavable (acidic, redox cleavage, and proteolytic). Eventually, carrier antibodies may include both antibody proteins and synthetic polymers. The discovery, analysis, and simulation of payload entities to linkers and carriers are impossible to be addressed in a traditional wetlab at this scale. Unquestionably, the only holistic option for the full studying and the in-depth analysis of all available CDR/framework properties of all antibodies in up-to-date biosciences is through modern bioinformatics. It is therefore, high time for a state-of-the-art and modern computational biology based approach to ADCs to be established. Thus, ensuring time and funding cost cutting and saving in tomorrows' eminent and emerging antibody-related drug design and drug delivery field.
